# Editorial Comment to Endoscopic laser treatment for urine leakage caused by an isolated calyx after robot‐assisted partial nephrectomy

**DOI:** 10.1002/iju5.12349

**Published:** 2021-09-12

**Authors:** Satoshi Kobayashi, Masaki Shiota

**Affiliations:** ^1^ Department of Radiology Brigham and Women's Hospital Harvard Medical School Boston MA USA; ^2^ Department of Urology Graduate School of Medical Sciences Kyushu University Fukuoka Japan

In this issue, Inoue et␣al. reported a case who was successfully treated with ureteroscopy using the holmium YAG laser to manage urinary leakage due to ligation of renal calyx during robot‐assisted partial nephrectomy (RAPN) for T1b renal tumor.^1^

RAPN for patient with T1b renal tumors has frequently been done without increasing the risk of complications.^2^ Preoperative image‐based assessment with R.E.N.A.L nephrometry score and PADUA nephrometry scores contribute to preventing complications.^3^ In addition, Mathieu et␣al. reported that surgeon's experience, blood loss, and opening urinary collecting system were important predictors of postoperative complications in RAPN.^4^ The frequency of urinary leakage after partial nephrectomy has been reported as between 0.8% and 5.2%.^5^ The resection with the renal calyx at the bottom of the tumor is inevitable to avoid positive surgical margins. Therefore, in RAPN for patients with the renal tumor close to collecting system, the risk of urinary leakage might increase due to defect of collecting system.

Ureteral stent, percutaneous nephrostomy, and percutaneous drainage should be considered for urinary leakage after RAPN refractory to conservative treatment. In this case, the 3‐0 V‐Loc^TM^ thread penetrated the renal calyx was visible on ureteroscopy, which lead to successful intervention. However, if the thread is invisible on ureteroscopy, alternative approach would be required; an intervention from outside the kidney such as laparoscopic procedure is necessary to release the stenosis of the renal calyx. Taken together, transurethral approach by retrograde pyelography and ureteroscopy plays an important role in the management of urinary leakage after partial nephrectomy, and should be considered as a first step when an intervention is required.

## Conflict of interest

The authors declare no conflict of interest.
